# Classification and Antioxidant Activity Evaluation of Edible Oils by Using Nanomaterial-Based Electrochemical Sensors

**DOI:** 10.3390/ijms24033010

**Published:** 2023-02-03

**Authors:** Irina Georgiana Munteanu, Constantin Apetrei

**Affiliations:** Department of Chemistry, Physics and Environment, Faculty of Sciences and Environment, “Dunărea de Jos” University of Galaţi, 47 Domneasca Street, 800008 Galaţi, Romania

**Keywords:** carbonaceous nanomaterials, sensor, extra virgin olive oil, data analysis, antioxidant activity

## Abstract

The classification of olive oils and the authentication of their biological or geographic origin are important issues for public health and for the olive oil market and related industries. The development of techniques for olive oil classification that are fast, easy to use, and suitable for online, in situ and remote operation is of high interest. In this study, the possibility of discriminating and classifying vegetable oils according to different criteria related to biological or geographical origin was assessed using cyclic voltammograms (CVs) as input data, obtained with electrochemical sensors based on carbonaceous nanomaterials and gold nanoparticles. In this context, 44 vegetable oil samples of different categories were analyzed and the capacity of the sensor array coupled with multivariate analysis was evaluated. The characteristics highlighted in voltammograms are related to the redox properties of the electroactive compounds, mainly phenolics, existing in the oils. Moreover, the antioxidant activity of the oils’ hydrophilic fraction was also estimated by conventional spectrophotometric methods (1,1-diphenyl-2-picrylhydrazyl (DPPH) and galvinoxyl) and correlated with the voltammetric responses of the sensors. The percentage of DPPH and galvinoxyl inhibition was accurately predicted from the voltammetric data, with a correlation coefficients greater than 0.97 both in calibration and in validation. The results indicate that this method allows for a clear discrimination of oils from different biological or geographic origins.

## 1. Introduction

The olive (*Olea europaea* L.) is an ancient crop in Mediterranean countries and has been spread throughout all five continents. The reason for its worldwide diffusion is related to the high value of its oils, which are used in human consumption and have been demonstrated to be highly beneficial for human health [1]. Olive fruits are very nutritious and their use together with the leaves in medicine dates back to ancient times [2]. Clinical data prove the beneficial properties of olive oil as a nutrient that reduces the risk of heart, skin or prostate diseases, as well as the risk of gastrointestinal cancer, etc., and it is included as an ingredient in numerous pharmaceutical and cosmetic formulations [3].

The assessment of virgin olive oil (VOO) quality and the classification into different categories is based on the following criteria: acidity, peroxide value, UV absorption and organoleptic evaluation [4]. On the basis of these criteria and taking into account the process of obtaining the various varieties of oil, the EU defines six main types of oils, which are delineated also by The International Olive Council (IOC) [5], in Article 118 of European Commission (EC) Regulation (EC) No 1234/2007 [6]. The specific characteristics of each type of oil are shown in Table S1.

The quality of the seed oils is established according to the Codex Standard for named vegetable oils 210 (Amendment 2005, 2011, 2013 and 2015) [7]. In the case of extra virgin sunflower oil, the technological process includes the cold extraction of the oil, which is suitable for human consumption without refining.

Of the many commercial categories that exist for olive oil, EVOO has particularly high standards in terms of composition as well as sensory characteristics as assessed by recognized panels [8]. Chemically, it is a complex food composed mainly of triglycerides, accounting for 98% of the total composition and a number of minor constituents that are of crucial importance in terms of health benefits and sensory properties [9]. This fraction of minor compounds includes free fatty acids [10], monoglycerides [11], diglycerides [12], phenols [13], tocopherols [14], sterols [15], phospholipids [16], waxes [17], squalene [17], hydrocarbons [18] and volatile compounds [19]. However, the phenolic compounds are the main compounds responsible for the flavor of EVOOs and as biomarkers of authenticity [20]. Furthermore, EVOO contains a high amount of phenolic compounds, which are the main agents responsible for resistance to auto-oxidation and photo-oxidation [21].

There are at least thirty-six structurally different phenolic compounds that have been identified in EVOO [22]. Not all of these compounds are present in every EVOO and there is variation in the concentration of phenolic compounds between olive oils [23]. Such variation in the phenolic fraction is the result of production factors, including the variety of olives [24], the region of olive cultivation [25], the agricultural techniques used for olive cultivation [26], the maturity of the olive fruit at harvest [27] and also the extraction, processing and storage methods [28]. The main phenolic compounds in olive oil are presented in Table S2.

The quantitative determination of these compounds is usually performed according to the Folin–Ciocalteu spectrophotometric method [29]. However, the method is less specific compared to chromatographic methods [30]. According to the literature, the most common analytical techniques used for the separation and determination of these phenolic compounds in olive oil to date are high-performance liquid chromatography (HPLC) coupled with ultraviolet (UV) or diode array detection (DAD) [31], mass spectrometry (MS) [32], gas chromatography coupled with MS [33], capillary electrophoresis (CE) coupled with UV or MS detection [34], nuclear magnetic resonance spectroscopy (NMR) [35], and infrared (IR) spectroscopy [36]. These methods have the disadvantage of being laborious [37], requiring a relatively long period of analysis and processing of the samples due to the complexity of the phenolic species [38]. Complementary innovative methods for the qualitative and quantitative determination of phenolic compounds are based on electrochemical sensors and biosensors [39,40,41]. The sensors and biosensors can be used for the detection of specific compounds or to obtain the chemical profile (“chemical fingerprint”) when an array of such sensors is used [42,43,44].

The array of sensors coupled with multivariate data analysis, electronic tongues, based on potentiometric, voltammetric or impedimetric sensors, have been successfully used for discriminating different types of oils, depending on their phenolic and sensory characteristics [45,46]. These features were used for the discrimination and classification of oils, and especially olive oils, as a function of quality, olive variety, bitterness or other sensorial characteristics, geographical origin, etc. [47,48].

An important category of electrochemical sensors and biosensors is based on carbonaceous nanomaterials and metal nanoparticles, which have a special affinity for the phenolic compounds [49]. These nanomaterials have high surface-to-volume ratios biocompatible with the phenolic compounds and enhance electron-transfer kinetics during electrochemical processes [50]. Therefore, these kinds of sensors and biosensors have excellent performance characteristics for the detection of phenolic compounds in complex samples.

In this study, screen-printed electrodes based on nanomaterials (carbon nanotubes, graphene and carbon nanofibers) modified with gold nanoparticles were used for the sensitive detection of phenolic compounds in oil hydromethanolic extracts. The synergic effects in the sensitive properties of nanomaterials increases the active area of sensors and improves the rate of electron transfer, increasing sensitivity. Electrochemical responses coupled with multivariate data analysis were used for discriminating and classifying vegetable oils according to different criteria, related to biological or geographical origin, or to establish correlations with antioxidant activity.

## 2. Materials and Methods

### 2.1. Reagents and Solutions

KCl was purchased from Sigma-Aldrich (St. Louis, MO, USA) and dissolved in ultrapure water obtained from a Milli-Q system (Millipore, Bedford, MA, USA). Methanol was purchased from Merck (Darmstadt, Germany).

The 0.1 mM DPPH stock solution was prepared by weighing 0.0036 g DPPH (2,2-diphenyl-1-picrylhydrazyl) reagent (purchased from Sigma-Aldrich) and dissolving in 100 mL 96% (*v*/*v*) ethanol (Sigma-Aldrich). The resulting solution was kept at room temperature and in the dark until use.

The 0.1 mM galvinoxyl stock solution was prepared by weighing 0.0042 g free radical galvinoxyl (2,6-di-tert-butyl-α-(3,5-di-tert-butyl-4-ox-2,5-cyclohexadiene-1-ylidene)-p-tolyloxy) reagent (purchased from Sigma-Aldrich) and dissolving in 100 mL 96% (*v*/*v*) ethanol, the mixture being kept in the dark at room temperature for 20 min before determination.

### 2.2. Electrodes and Devices Used

Electrochemical measurements were performed using a conventional system containing three electrodes, namely an Ag/AgCl reference electrode (Princeton, Applied Research), an auxiliary electrode consisting of a platinum wire, and a working electrode. The working electrode was, in turn, a screen-printed electrode (SPE) based on nanomaterials such as carbon nanotubes (CNT) and gold nanoparticles (GNP), nanofibers (CNF) and GNP and also, graphene (GPH) and GNP. These three working electrodes were purchased from Metrohm-DropSens (Llanera, Spain).

A Biologic SP 150 potentiostat/galvanostat (Bio-Logic Science Instruments SAS France) coupled with EC-Lab Express software operating in Windows was used to record, characterize and optimize the electrode signals. Software was also used to analyze and interpret the results: Origin, The Unscrambler and Microsoft Excel. For the weighting of the compounds, the Partner AS 220/C/2 analytical balance was used, as well as the Elmasonic ultrasonic bath (Carl Roth GmbH, Karlsruhe, Germany) for the dissolution and homogenization of the solutions.

For the spectrophotometric method based on the reaction of antioxidants with the stable free radicals DPPH and galvinoxyl, sample absorbances were measured using a Rayleigh UV2601 UV/Vis Double Beam Spectrophotometer (Beijing Beifen-Ruili Analytical Instrument, Beijing, China).

### 2.3. Samples

44 different commercial oils with year of production 2022 were analyzed in this study, divided into three types of edible vegetable oil, namely POO, EVOO from various countries (Italy, Greece, Spain and Tunisia), and unrefined sunflower oils (SFO). All oils studied were purchased from a local supermarket. A sub-criterion for EVOO sample classification was the country of origin. The studied samples were packaged in dark 0.5 or 1 L bottles and their price was not higher than 8 €/L at the time of the study. Oil samples were kept in a dry and dark place, at a constant temperature. Table 1 summarizes all the samples of edible oils studied.

### 2.4. Obtaining Extracts

For analysis, 44 samples from different oils were prepared using liquid–liquid extraction [51]. For each oil, 5 g was mixed with 10 mL methanol-water solution (40:10, *v*/*v*) and ultrasonicated for 10 min. The hydromethanolic extracts were separated using a separation funnel. For the measurements with the sensors, 5 mL of hydromethanolic extract was added to 45 mL 10^−1^ M KCl solution and the CVs were registered. Six replicates for all samples and sensors were registered. These data were used as input in multivariate data analysis.

### 2.5. DPPH Method

The DPPH assay for the evaluation of antioxidant activity is a simple, cheap and effective method, one of the most commonly used to determine the antioxidant capacity of a compound, an extract or other biological matrices (plants, fruit, wine, honey) [52,53,54]. In its initial radical form, DPPH has an intense purple color, which changes to yellow when found in reduced form (Figure S1).

DPPH has a significant absorption band in the range 515–520 nm, making spectrophotometry an easy tool for measuring this color change and determining the antioxidant activity of the sample. The more this color changes, the more DPPH is reduced and the better the antioxidant activity of the sample [55]. The use of the DPPH assay provides an easy and rapid way to assess antioxidants by spectrophotometry, and various chemical compounds or natural products with antioxidant activity can be evaluated [56].

### 2.6. Galvinoxyl Method

The galvinoxyl method of determining antiradical activity is based on the use of the stable O-centered radical galvinoxyl, which is known to associate with the physiological action of oxygen radicals rather than the stable N-centered radical of DPPH [57]. Galvinoxyl exhibits strong absorption at 860 nm that is used for the spectrophotometric determination of the antioxidant activity based on a decrease in the absorbance intensity in the presence of antioxidants [58]. The same sensing mechanism of radical scavenging described in Figure S1 can be applied to this radical. However, the color change induced by exposure to antioxidants is not easily detected by the naked eye. The galvinoxyl radical-scavenging reaction is given in Figure S2.

### 2.7. Data Analysis

The input data used in the multivariate data analysis were the CVs obtained with three electrochemical sensors immersed in the hydromethanolic extracts of all oils under study. Six replicates CVs of all electrochemical sensors were registered for all the oil sample hydromethanolic extracts and all CV data were used as input in the multivariate data analysis.

The cyclic voltammograms were registered between −0.4 and 1.3 V and the scan rate was 0.1 V·s^−1^. No signal pretreatment was applied. All CVs were imported in Excel and the constant variable (potential values) was removed. The current values of all replicates and samples from the anodic scan were included in the input matrix. From the analysis of the CVs it was observed that the main differences between the samples was in the anodic part. In order to respect the convention that in the data matrix the samples must be the lines and the variables the columns, the matrix containing the anodic current values was transposed. In this way, a matrix with 264 rows (44 samples × 6 replicates) and 480 columns (3 sensors × 160 current values) for the anodic part of CV was obtained.

Multivariate data analysis was performed to determine if there were significant differences between the oils studied and for their classification as a function of type of oil, or country of origin (in the case of EVOOs).

Principal component analysis (PCA) was used to reduce the variables and discriminate into different classes. PCA is a multidimensional analysis that allows the transformation of the original variables into new ones, called principal components. The role of principal components is to explain the maximum amount of variance with the smallest number of components [59,60].

PLS-DA (partial least squares—discriminant analysis) has been used as a deterministic classification technique. The basis of PLS-DA consists primarily of applying a partial regression model using the least squares method on variables that are indicators of groups. The second step of PLS-DA is to classify the observations from the PLS regression results on the indicator variables [61]. By projecting intercorrelated data from a high-dimensional space into a low-dimensional orthogonal space, the newly formed variables, which are linear combinations of the original variables, become orthogonal to each other [62]. By finding the discriminant plane to effectively separate data into different classes, PLS-DA is capable of separating “tight” classes of observations on the basis of the X-variables (CVs of the sensors), according to a Y-vector that encodes the class membership in a set of categorized variables, denoted as positive and negative (1 and 0 values, respectively) [63].

Partial Least Squares regression (PLS) is a method which reduces the variables used to predict to a smaller set of predictors, which is then used to perform a regression [64]. PLS 1 corresponds to the case where there is only one dependent variable. The PLS1 regression model was used to establish correlations between electrochemical sensor responses and antioxidant activity determined by the DPPH spectrophotometric method or galvinoxyl method (% inhibition degree is the dependent variable).

Multivariate data analysis was performed using Matlab, Excel and Unscrambler.

## 3. Results and Discussion

### 3.1. Electrochemical Study of Oil Extracts with Nanomaterials-Based Sensors

To study electrochemical processes, reversibility of reactions and stability of the response of the three sensors, the CV electrochemical method was used. The determinations were carried out in the potential range between −0.4 and 1.3 V and the scan rate was 0.1 V·s^−1^.

**Figure 1:**
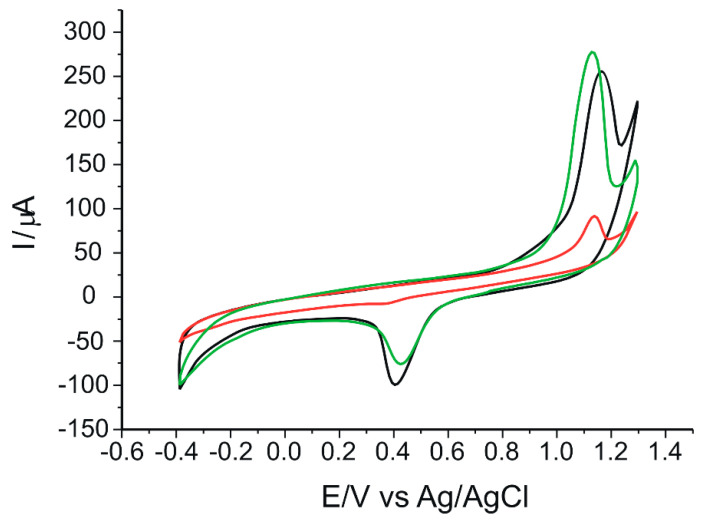
Voltammetric responses of CNT-GNP/SPCE (red line), CNF-GNP/SPCE (black line) and GPH-GNP/SPCE (green line) towards extract of EVOO sample v1. Scan rate: 0.1 V·s^−1^.

In the first step, the behavior of the three electrodes CNT-GNP/SPE, CNF-GNP/SPE and GPH-GNP/SPE immersed in the extract solutions was studied. Figure 1 shows the CVs of the three sensors when immersed in the same extract from EVOO sample v1.

It can be seen that each sensor has a particular response when immersed in a solution of the same extract. These differences among the responses of the three electrodes is attributed to the redox activity of the phenolic compounds present in the sample to be analyzed [65], the oxidation and reduction processes being facilitated differently by the modifying nanomaterials present in the sensitive elements of the sensors [66,67].

It was also observed that the highest intensity at which the oxidation peak occurs is obtained in the case of GPH-GNP/SPCE (276.22 µA), while the intensity at which the reduction peak occurs is higher in the case of CNF-GNP/SPCE (−98.16 µA). These increases in oxidation and reduction current intensity could be attributed to the association of nanomaterials CNT, GPH and also CNF with GNP, achieving a synergistic effect and contributing to increase the conductivity of the electrodes and the electron transfer rate, thus increasing the sensitivity of the sensors [68,69]. Therefore, the three sensors immersed in the same extract solution provide complementary chemical composition information and the CVs are chemical fingerprints of the analyzed samples.

**Figure 2:**
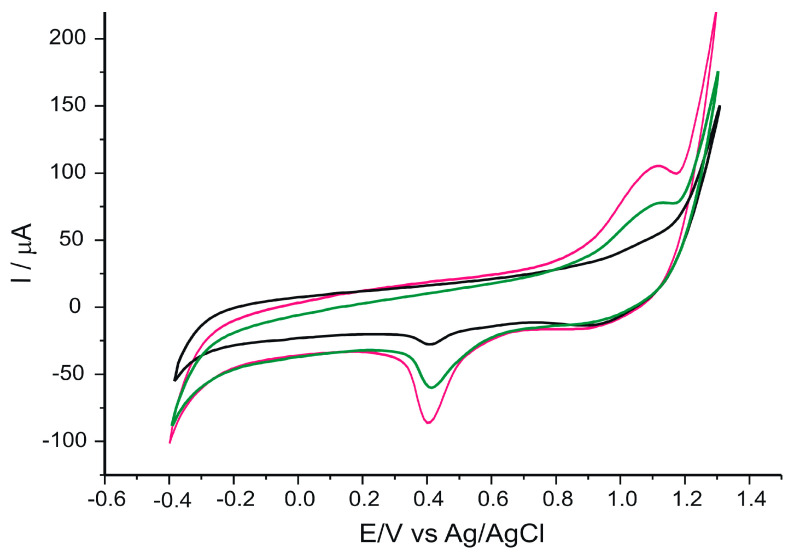
CVs of CNF-GNP/SPCE sensor when immersed in EVOO sample v2 (red line), POO sample p2 (green line) and SFO sample s2 (black line). Scan rate 0.1 V·s^−1^.

To highlight the differences occurring between extracts from different samples, the following figure shows the response of the CNF-GNP/SPCE sensor when immersed in EVOO sample v2, POO sample p2 and SFO sample s2 (Figure 2).

Important differences in peak intensity, position and shape were observed from one sample to another, depending mostly on the polyphenolic compound content of the respective samples. At the same time, a shift of oxidation peak potentials towards lower values is observed with increasing polyphenolic compounds content [70]. The results shown in Figure 2 indicate that the nature and concentration of the compounds found in different classes of oils give rise to a variety of electrochemical signals that can be used to discriminate between different types of oils. In particular, olive oils can be easily distinguished from seed oils due to their high polyphenol content [71].

**Figure 3:**
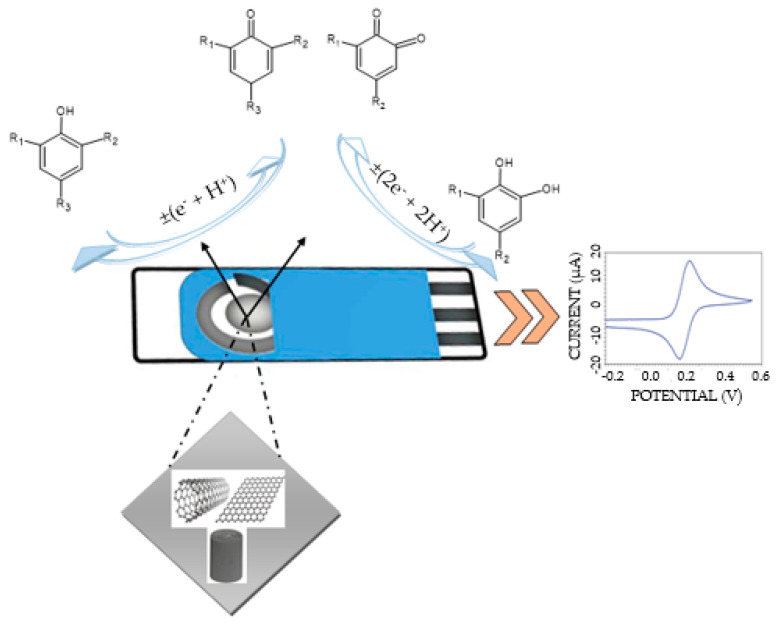
Schematic representation for the mechanism of mono- and di-phenolic compound detection using the electrochemical method.

Figure 3 shows the detection mechanism of mono- and di-phenolic compounds in the samples studied with voltammetric sensors. A single redox process is observed, involving the exchange of one electron and one proton in the case of mono-phenolic compounds, and the exchange of two electrons and two protons in a single step in the case of di-phenolic compounds, with the formation of the respective quinone derivative in both cases.

The nanomaterials from the sensitive element of the sensors facilitate the detection of phenolic compounds from the samples, the CVs being well defined, with reduced noise and high current peaks. The peak potentials and currents are related to the nature of the analyzed samples.

### 3.2. Discrimination and Classification of Oils Using Multivariate Data Analysis

**Figure 4:**
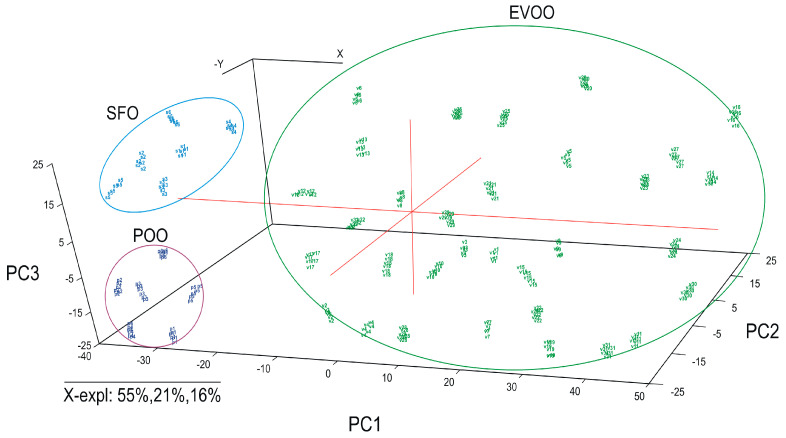
PCA scores plot corresponding to the CV data corresponding to different categories of oils: EVOO, POO and SFO samples.

**Figure 5:**
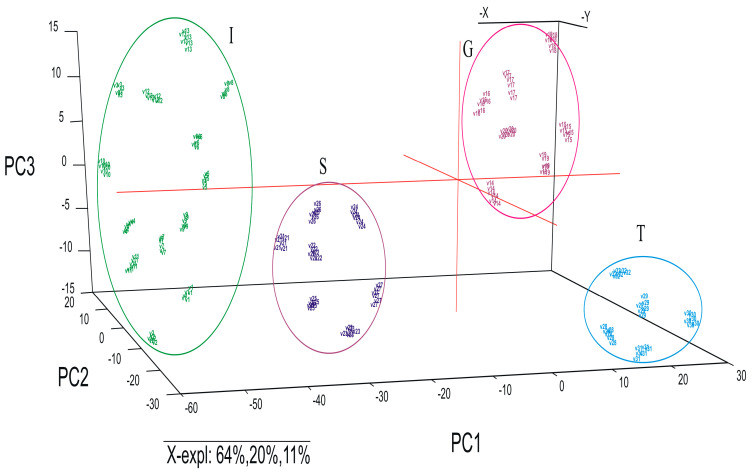
PCA scores plot corresponding to the CV data obtained with the sensors when these are exposed to EVOO samples from Italy, Spain, Greece and Tunisia.

In the first step, electrochemical data from the input matrix were used to perform the PCA. The data from the input matrix were firstly pre-processed by centering the variables and scaling them to unit variance. The purpose of this treatment was to give all variables included in PCA an equal chance to influence the model, regardless of their original variance. The normalization was performed with the 1/(Standard Deviation) method. For this data analysis, the entire data matrix was split into two different data sets including the specific samples and all the corresponding variables differentiated by different criteria: type of the oils (all samples) and the country of origin (all the EVOO samples). The results are presented as three-dimensional score plots, differentiated by EVOO, POO or SFO—all samples were included in the model (Figure 4), and by country of origin—Italy, Greece, Spain and Tunisia—for all the EVOO samples (Figure 5).

The graph in Figure 4 shows that the system based on the three sensors immersed in different solutions of hydromethanolic extracts is able to discriminate EVOO from POO and SFO samples, with the first three main components explaining 92% of the variation. Thus, the first principal component explains 55% of the variance of the electrochemical signal, the second principal component accounts for 21% and the third principal component for 16%. It can be seen that EVOO appeared in the right region of the score plot (mostly at positive PC1 values), while POO appeared on the left side of the plot (at negative PC1 values). SFO also appeared at the negative PC1 values, but was well discriminated from other types of oils. PC2 is relevant for the discrimination between POO and SFO. Furthermore, PC2 is important for the discrimination of the samples of each category. As can be observed, the three groups are very well separated and this indicates that the samples could be clearly discriminated using the electrochemical sensors array based on nanomaterials.

Another principal component analysis was carried out to explore the structure of the data and the possibility of discriminating EVOO samples by country of origin. The scores plot of PCA based on the CVs of all voltammetric sensors immersed in the extracts of EVOOs is presented in Figure 5.

In the PCA score plot, four distinct groups of EVOOs could be found, grouped according to country of origin (Figure 5). The members of the first group on the left side of the scores plot are olive oil samples from Italy. This group is well separated from the second group consisting of samples from Spain. The samples from Greece and Tunisia are separated from the other two categories and are found on the right side of the plot (at positive values of PC1). PC2 is very important in the discrimination of the EVOOs from Greece and Tunisia. All three PCs, which explain 95% of the variance, have the highest importance in the discrimination of the samples in agreement with EVOO country of origin, indicating that the geographical location had a great influence on the chemical composition of the olive oils.

The PLS-DA technique, a supervised classification method that allows verification of the assignment of a sample to a particular group and calculation of the calibration and validation error, was used to confirm the groups observed in the principal component analysis.

PLS-DA modeling involves two main procedures, PLS-DA component construction (dimension reduction) and prediction model construction (discriminant analysis). The output of the PLS-DA algorithms is the X-score (PLS-DA scores), which represents the original data X in a lower-dimensional subspace, and the predicted class membership matrix (Y_pred_), which estimates the class membership of the samples [64]. PLS-DA is used to optimize separation between different groups of samples, which is accomplished by linking two data matrices X (sensor data) and Y (classes of samples).

PLS-DA aims to maximize the covariance between the independent variables X (sensors data) and the corresponding dependent variable Y (classes of samples) of highly multidimensional data by finding a linear subspace of the explanatory variables [62]. This new subspace permits the prediction of the Y variable based on a reduced number of factors (latent variables, LV). These factors describe the behavior of dependent variables Y and they span the subspace onto which the independent variables X are projected [63]. For example, if the samples are divided into two different classes, then the variable Y will comprise a single vector, which will have an entry of 0 for all samples in the first class and an entry of 1 for all samples in the second class. When the data contain three classes, then the three groups will be binarily encoded in 3 variables with the Y matrix as [1 0 0] for all samples from class A, [0 1 0] for samples from class B, and finally [0 0 1] for samples from class C. This is the case for PLS-DA taking into account the type of oil: EVOO, POO and SFO.

**Figure 6:**
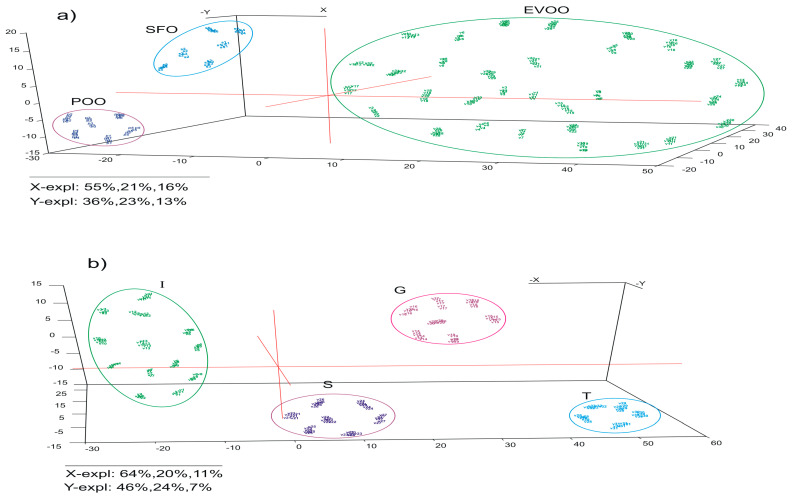
PLS-DA scores plot resulting from the sensor data (X matrix) corresponding to different classes of oils (Y matrix): (**a**) EVOO, POO and SFO samples; (**b**) Italy, Spain, Greece and Tunisia EVOO samples.

Figure 6 shows the PLS-DA scores plots developed from the same data used for PCA (X matrix), and the dependent variables were the classification criteria observed in PCA scores plots (Y matrix): type of oil (Figure 6a) and country of origin (Figure 6b), respectively. The random test cross-validation method was used to validate the models. In this method, the training and test samples are randomly selected. The random test was performed for 100 runs by removing 2 randomly selected samples. The optimal number of latent variables (LV) was determined according to the minimum predicted residual error sum of squares.

In Figure 6, it can be seen that the distribution of the samples in the scores plots is similar to that observed in the case of the PCA scores plots. However, a greater separation of the sample groups based on different criteria was observed.

The quantitative data of all PLS-DA models are collected in Table 2, with correlation coefficients greater than 0.97 and root mean square errors very low at both calibration and validation.

From these results, it can be concluded that the electrochemical data obtained with the voltammetric sensors based on different carbonaceous nanomaterials and gold nanoparticles can be successfully used for the classification of the oils based on different criteria such as type of oil for EVOO, POO and SFO samples, or country of origin for EVOO samples.

### 3.3. Determination of Antioxidant Activity. Correlation between Sensor Response and Spectrophotometric Measurements to Determine Antioxidant Activity

An evaluation of antioxidant activity with the DPPH spectrophotometric method was performed according to the method previously described by Gali et al. [72]. The assay was carried out by adding volumes of 1 mL extract solution to 3 mL 0.1 mM DPPH ethanolic solution in the spectrophotometer cuvettes and then measuring the absorbance at λ = 517 nm towards ethanol.

For the application of the galvinoxyl method for the analysis of oil samples, volumes of 1 mL extract solution were added to 3 mL 0.1 mM galvinoxyl solution in the spectrophotometer cuvettes and the absorbance for each sample was then measured at 860 nm towards ethanol after 20 min [73].

For both methods, the percentage of DPPH inhibition and the percentage of galvinoxyl inhibition were calculated according to the following equation [74]:

$$\%\mathrm{Inhibition}=\left(\frac{\mathrm{AD}-\mathrm{AE}}{\mathrm{AD}}\right)\times 100$$

where AD is the absorbance of the control solutions and AE is the absorbance of the test solutions.

The % inhibition results obtained by both methods are shown in Table 3. All the analyses were carried out in triplicate and the average values are reported. The standard deviation of the measurements was lower than 2% for all the measurements.

As can be seen from the table above, the percentage inhibition values are mostly close in value, but the highest inhibition percentage was obtained for Costa d’Oro L’Italiano EVOO. This result can be correlated with a higher intensity of the anodic peak and, therefore, with a higher content of polyphenolic compounds.

The next step of the study was to establish correlations between the electrochemical signals provided by the sensors and the data obtained from the spectrophotometric determination of antioxidant activity by the DPPH and galvinoxyl methods.

PLS1 was used as a prediction technique to correlate the voltammetric data obtained with the sensors with the antioxidant activity quantified as capacity of free radical scavenging. The independent variables (X matrix) were all CVs obtained with the sensors and the dependent variable was the percentage of DPPH inhibition or the percentage of galvinoxyl inhibition, respectively. The normalization of the data was performed with the 1/(Standard Deviation) method.

The results of PLS1 regression models are presented in the form of the dependences between predicted antioxidant activity and measured antioxidant activity.

**Figure 7:**
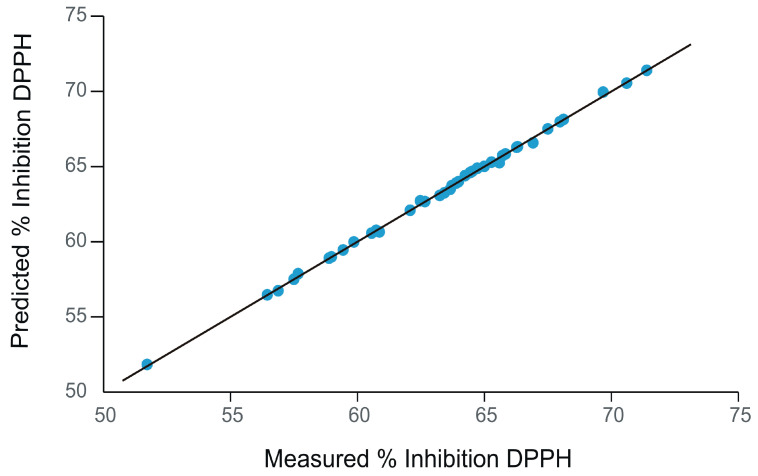
Plot of predicted % inhibition of DPPH from the voltammetric data vs. measured % inhibition of DPPH obtained from the spectrophotometric methods. The averages of six replicates corresponding to each oil sample were included in the regression models.

Figure 7 shows the dependence plot between the percentage inhibition value of DPPH free radical predicted from voltammetric data and the values measured by the spectrophotometric method.

As can be observed in Figure 7, the voltammetric data obtained with the sensors are able to predict the antioxidant properties of the samples expressed as the capacity to scavenge the free radical DPPH with good accuracy. Similar results were obtained for the galvinoxyl test.

Table 4 shows the quantitative data obtained from the CV-DPPH and CV-galvinoxyl PLS1 regression models. It can be noted that both calibration and validation values indicate good model performance (correlation coefficient close to 1). In addition, low values of the root mean square error at calibration (RMSEC) and root mean square error at prediction (RMSEP) were obtained for both PLS1 models.

From the data included in Table 4, it can be seen that, from the data obtained with the electrochemical sensors by CV, it is possible to accurately estimate the antioxidant activity of the oils expressed as the inhibition capacity towards the free radicals DPPH and galvinoxyl, respectively.

## 4. Conclusions

In this study, models were developed for discriminating and classifying oil samples based on different criteria, using an electrochemical method based on SPE modified with different carbonaceous nanomaterials and GNP. The electrochemical signals were used as input variables in multivariate data analysis studies. At the same time, it was established that there was a very good correlation between the electrochemical responses of the sensors when immersed in solutions from the 44 oil samples studied and the data obtained from the DPPH and galvinoxyl spectrophotometric assays for the assessment of antioxidant activity. As a result of this study, it can be concluded that electrochemical sensors can be useful for the estimation of antioxidant activity of different types of oils, including EVOO, but also that they can be used for the discrimination and classification of vegetable oils.

The use of sensors and biosensors in industry and quality control laboratories is still limited, even though these have numerous advantages and adequate sensitivity. Further studies will include biosensors capable of detecting biomarkers that could improve the performance of the analytical system for discrimination, classification or authentication. Thus, the development of databases using analytical information obtained by electroanalytical methods, together with the use of appropriate statistical tools could be useful in increasing the performance of systems for the quality control of EVOOs. Another research perspective would be the development of a lab-on-a-chip device useful in the routine analysis of olive oil at different stages of production, from harvesting to marketing, and also to analyse oxidative stability.

## Figures and Tables

**Table 1:** Oil denomination, code, type of oil, and country of origin for the 44 oil samples under study.

Sample	Oils Denomination	Code		
			Type of Oil	Country of Origin
1	TopSeller Pomace Oil	p1	POO	-
2	Oliol	p2	POO	-
3	Costa d’Oro, Sansa	p3	POO	-
4	Regina Olio di Sansa	p4	POO	-
5	Pietro Coricelli Olio di Sansa	p5	POO	-
6	Ionia	p6	POO	-
7	Regina	v1	EVOO	I
8	Costa D’Oro L’extra	v2	EVOO	I
9	Olitalia	v3	EVOO	I
10	Pietro Coricelli Non Filtrato	v4	EVOO	I
11	Mazza	v5	EVOO	I
12	Rivano Olio	v6	EVOO	I
13	Costa d’Oro Il Grezzo	v7	EVOO	I
14	Pietro Coricelli Olio	v8	EVOO	I
15	Monini Mini	v9	EVOO	I
16	Costa d’Oro L’Italiano	v10	EVOO	I
17	Costa d’Oro, Il Biologico	v11	EVOO	I
18	Monini Delicato	v12	EVOO	I
19	Monini Classico	v13	EVOO	I
20	Solaris Koroneiki	v14	EVOO	G
21	Greek Koroneiki	v15	EVOO	G
22	Minerva Greek	v16	EVOO	G
23	Monastir Oil	v17	EVOO	G
24	Kanakis	v18	EVOO	G
25	Agoureleo	v19	EVOO	G
26	Agoureleo Finest	v20	EVOO	G
27	Mueloliva	v21	EVOO	S
28	TopSeller Extra Virgin Olive Oil	v22	EVOO	S
29	Molino Alfonso	v23	EVOO	S
30	Pletora	v24	EVOO	S
31	Iznaoliva	v25	EVOO	S
32	Coosur	v26	EVOO	S
33	Obio	v27	EVOO	S
34	Terra Delyssa—Tunisian Oil	v28	EVOO	T
35	Clearspring	v29	EVOO	T
36	Urtekram	v30	EVOO	T
37	Terra Delyssa Bio	v31	EVOO	T
38	Olivi	v32	EVOO	T
39	Solaris	s1	SFO	-
40	De la Luna	s2	SFO	-
41	Walachia	s3	SFO	-
42	Morarita	s4	SFO	-
43	Bunetto	s5	SFO	-
44	Super Foods	s6	SFO	-

EVOO—extra virgin olive oil; POO—pomace olive oil; SFO = sunflower oil; I = Italy; G = Greece; S = Spain; T = Tunisia.

**Table 2:** Quantitative data of the PLS-DA regression using voltammetric data for two classification models.

Classification Criterium	Calibration				Validation			
	Slope	Offset	R_C_	RMSEC	Slope	Offset	R_P_	RMSEP
**Type**								
EVOO	0.993	0.008	0.989	0.064	0.988	0.011	0.988	0.074
SFO	0.974	0.010	0.973	0.029	0.971	0.009	0.971	0.044
POO	0.985	0.005	0.983	0.016	0.981	0.006	0.980	0.041
**Country of origin**								
Italy	0.993	0.009	0.992	0.022	0.991	0.011	0.986	0.048
Greece	0.990	0.012	0.986	0.044	0.989	0.010	0.980	0.063
Spain	0.994	0.004	0.990	0.032	0.992	0.006	0.984	0.054
Tunisia	0.991	0.007	0.989	0.026	0.988	0.005	0.981	0.052

R_c_—correlation coefficient of calibration; R_p_—correlation coefficient of prediction; RMSEC—Root Mean Square Error of Calibration; RMSEP—Root Mean Square Error of Prediction.

**Table 3:** Antioxidant activity of oil samples studied expressed as % inhibition of the free radicals DPPH or galvinoxyl.

Sample	% Inhibition DPPH	% Inhibition Galvinoxyl	Sample	% Inhibition DPPH	% Inhibition Galvinoxyl
1	65.00	23.53	23	64.72	23.53
2	58.89	13.73	24	62.44	19.47
3	60.83	15.79	25	63.72	20.45
4	56.45	13.45	26	64.24	20.35
5	57.50	12.76	27	66.94	27.45
6	59.44	16.98	28	65.83	25.89
7	60.56	13.73	29	66.31	26.64
8	63.89	18.65	30	67.98	27.53
9	51.67	7.76	31	66.27	25.98
10	65.28	25.49	32	65.71	27.41
11	64.72	21.97	33	68.12	25.54
12	63.89	17.75	34	63.33	17.65
13	67.50	31.37	35	64.52	18.54
14	64.44	19.00	36	62.66	16.43
15	64.72	22.57	37	63.98	17.61
16	71.39	36.18	38	62.08	17.21
17	65.56	25.69	39	60.56	15.69
18	70.83	34.33	40	59.84	17.65
19	69.72	33.33	41	56.88	20.67
20	63.56	20.43	42	57.65	19.96
21	63.15	16.76	43	58.98	16.82
22	64.72	21.57	44	60.74	16.45

**Table 4:** Results of PLS1-DPPH and PLS1-galvinoxyl regression models in calibration and validation.

	PLS1 DPPH Regression Model	PLS1 Galvinoxyl Regression Model
**Calibration**		
Slope	0.963	0.959
Offset	2.752	0.984
Correlation	0.984	0.991
RMSEC	1.560	1.412
**Validation**		
Slope	0.953	0.951
Offset	3.104	1.016
Correlation	0.975	0.984
RMSEP	1.628	1.712

## Data Availability

Not applicable.

## Supplementary Materials

The following supporting information can be downloaded at: https://www.mdpi.com/article/10.3390/ijms24033010/s1.
