# Supplementary material for: Classification and Antioxidant Activity Evaluation of Edible Oils by Using Nanomaterial-Based Electrochemical Sensors
Source: Int J Mol Sci. 2023 Feb 3;24(3):3010. doi: 10.3390/ijms24033010 (PMC9917972; doi:10.3390/ijms24033010)
Supplement: Supplementary file 1 [file ijms-24-03010-s001.zip › ijms-2180135-supplementary.pdf]

International Journal of *Molecular Sciences*

MDPI

Supplementary materials

Classification and Antioxidant Activity Evaluation of Edible Oils by Using Nanomaterials-Based Electrochemical Sensors

Irina Georgiana Munteanu and Constantin Apetrei *

Department of Chemistry, Physics and Environment, Faculty of Sciences and Environment, "Dunărea de Jos" University of Galați, 47 Domneasca Street, 800008 Galați, Romania
* Correspondence: apetreic@ugal.ro; Tel.: +40-727-580-914

Table S1. Types of olive oils. Modified from EU Council Regulation (EC) No 1234/2007.

Type of Oil		Characteristics	Free Acidity
**Virgin Olive Oils**	EVOO	They are obtained by mechanical or other physical processes under specific thermal conditions that do not cause alterations in the oil and have not suffered any treatment other than washing, decantation, centrifugation or filtration. Excluded are oils obtained using solvents or adjuvants with chemical actions, by re-esterification process or any mixture with oils of other types.	<0.8 g per 100 g
	VOO		≤2 g per 100 g
	Lampante olive oil		>2 g per 100 g
Refined olive oil		To obtain this type of oil, VOO is submitted to a refining process.	≤0.3 g per 100 g
Olive oil (composed of refined olive oils and virgin olive oils)		It is the result of the blending of EVOO and VOO with refined olive oil.	≤1 g per 100 g
Crude POO		This is the oil obtained from olive pomace by using solvents, physical treatments or oil corresponding to lampante olive oil type, except for certain specified characteristics. As well as in the case of virgin olive oils, excluded are oils obtained by means of re-esterification and mixtures with oils of other types	
Refined POO		This type is obtained from refining crude POO	≤0.3 g per 100 g
POO		This represents the resultant oil from mixing refined POO and VOO different than lampante oil.	≤1 g per 100 g

EVOO – extra virgin olive oil; POO - pomace olive oil.

Table S2. Phenolic compounds present in EVOO.

**Benzoic acid derivatives**	**R_1**	**R_2**	**R_3**	**R_4**	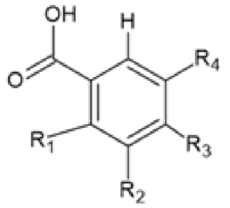
Benzoic acid	H	H	H	H	
p-Hydroxybenzoic acid	H	H	OH	H	
Vanillic acid	H	OCH_3	OH	H	
Gallic acid	H	OH	OH	OH	
Syringic acid	H	OCH_3	H	OCH_3	
Protocatechuic acid	H	OH	OH	H	
**Cinnamic acid derivatives**	**R_1**	**R_2**	**R_3**	**R_4**	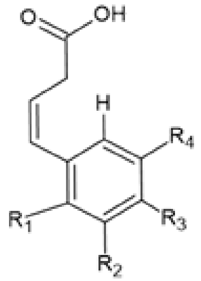
Cinnamic acid	H	H	H	H	
o-Coumaric acid	OH	H	H	H	
m-Coumaric acid	H	OH	H	H	
p-Coumaric acid	H	H	OH	H	
Ferulic acid	H	H	OH	OCH_3	
Caffeic acid	H	OH	OH	H	
Sinapic acid	H	OCH_3	OH	OCH_3	
**Phenolic alcohols**	**R_1**	**R_2**	**R_3**	**R_4**	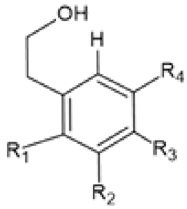
Hydroxytyrosol	H	H	OH	OH	
Tyrosol	H	H	OH	H	
**Secoiridoids**	**R_1**	**R_2**	**R_3**	**R_4**	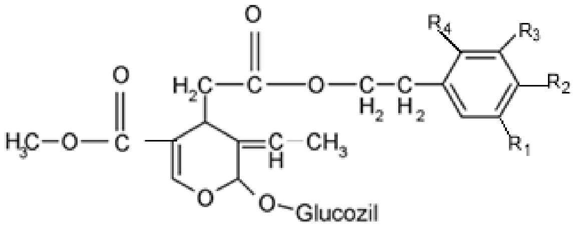
Oleuropein	H	OH	OH	H	
Ligstroside	H	OH	H	H	
**Flavonoids - Flavones**	**R_1**	**R_2**	**R_3**	**R_4**	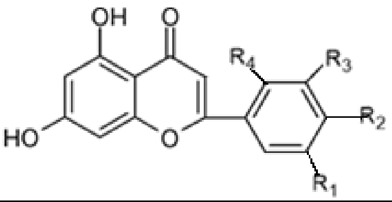
Apigenin	H	OH	H	H	
Luteolin	H	OH	OH	H	

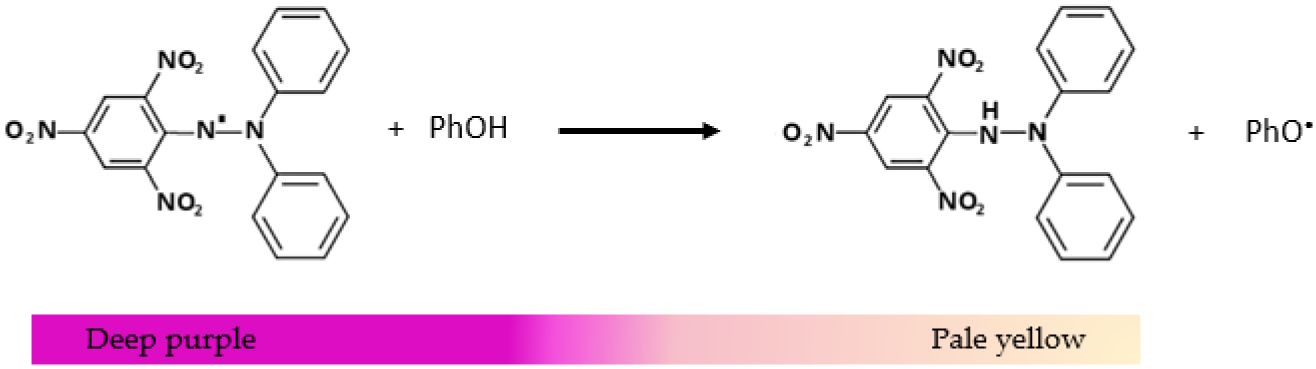

Figure S1. DPPH scavenging mechanism by an antioxidant (PhOH = phenolic compound).

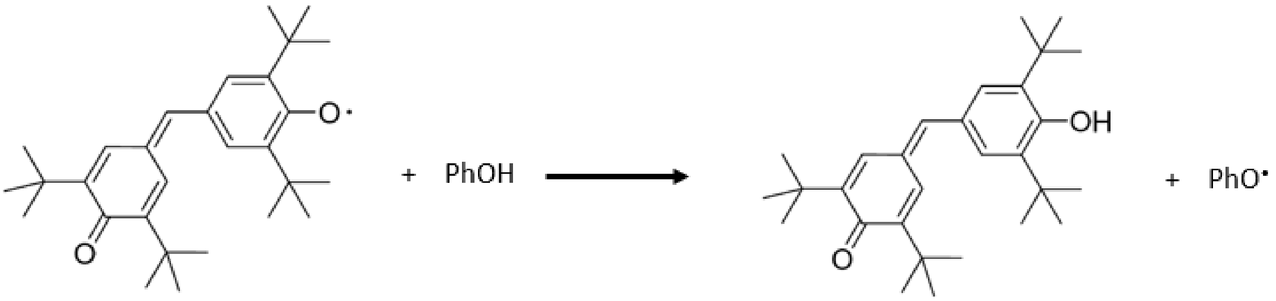

Figure S2. Galvinoxyl scavenging mechanism by an antioxidant (PhOH = phenolic compound).
